# Design and application of a new 24-h water sampler for monitoring particulate and dissolved road pollutants

**DOI:** 10.1007/s10661-025-14671-6

**Published:** 2025-10-10

**Authors:** Katie McKenzie, Angela Pllu, Iain Campbell, Siobhan Anderson, Linda A. Lawton, Bruce Petrie

**Affiliations:** 1 School of Pharmacy, Applied Sciences and Public Health, Robert Gordon University, Aberdeen, AB10 7GJ UK; 2 Balfour Beatty plc, UK Construction Services – Motherwell, North Lanarkshire, ML1 4WQ UK; 3 The Tyre Collective LTD, Royal Institution, 21 Albemarle Street, London, W1S 4BS UK

**Keywords:** Tyre, HMMM, Sustainable urban drainage system, Microplastic, TRWP, Paint

## Abstract

Road pollution is a threat to aquatic ecosystems globally. Road runoff contains a mixture of particulate (e.g. tyre and road wear particles (TRWPs), road lining paint fragments and microplastics) and ‘dissolved’ pollutants (e.g. leached tyre and plastic additive chemicals). There is a lack of sampling approaches, however, for collection of particulate and dissolved pollutants which avoids transport of large volumes of water needed for particulate analysis and minimises sample contact with plastic materials. Therefore, the aim was to develop a new stainless-steel 24-h portable sampler for in situ isolation of particulates from a large sample volume (> 10 L) and simultaneous collection of water for additive chemical analysis. This was achieved using readily available materials (battery powered peristaltic pump, stainless-steel refuse vessel, sieves etc.) and at < 50% of the cost of commercially available plastic bodied composite samplers. The newly designed sampler was applied in the field to monitor pollutants entering a retention pond from a road drainage system. Particle concentrations > 50 µm in length were 25.5, 13.7 and 2.0 particles/L for TRWPs, paints and microplastics, respectively, with most (89%) in the 50–99-µm size range. Five additive chemicals were also determined in the collected water (1H-benzotriazole, 5-methylbenzotriazole, hexamethoxymethylmelamine, 1,3-diphenylguanidine and 1-cyclohexyl-3-phenylurea) at concentrations up to 0.18 µg/L. This new sampler has demonstrated to be effective for the simultaneous monitoring of particulate and dissolved road pollutants in water. Its ease of construction, limited plastic usage and low cost make it an attractive alternative to existing sampling methods for monitoring road pollution.

## Introduction

Pollution from roads can impact aquatic ecosystems (Sun et al., 2018). Recent research has identified tyre and road wear particles (TRWPs), road lining paint fragments, microplastics and tyre and plastic additive chemicals as emerging pollutants of concern (Goehler et al., 2022; Liu et al., 2019; Rauert et al., 2022a, 2022b; Worek et al., 2022). There is a growing body of evidence on their presence in road runoff and in engineered systems to manage road runoff (e.g. retention ponds) (Goehler et al., 2022; Liu et al., 2019; Ziajahromi et al., 2020). However, there is no single accepted method of sampling water for particulate (e.g. TRWPs, paints and microplastics) and ‘dissolved’ road pollutants (e.g. tyre and plastic additive chemicals) which is problematic as findings are affected by the sampling process (Huang et al., 2023). Grab samples only provide information for that moment in time, and short-term pollutant variations are not appreciated. High-frequency grab sampling is usually not possible due to the often-laborious nature of particulate analysis. This can be overcome by 24-h composite sampling. Commercially available portable options normally have plastic collection vessels which increase the risk of introducing unwanted plastic contamination. However, if glass collection bottles are not available, they can usually be retrofitted. Nevertheless, both grab and 24-h composite water samples (often ≥ 10 L for particulate analysis) need transported back to the laboratory for processing (Hou et al., 2019; Peter et al., 2018; Rauert et al., 2022a, 2022b; Ziajahromi et al., 2017). This can be challenging due to the weight of sample and suitable sample vessels (e.g. glass).

Ziajahromi et al. (2017) developed an innovative sampler whereby water is passed through a series of sieves to collect particles in situ which avoids the need to transport large volumes of water. This can be used as a continuous sampling procedure for several hours (depending on the heterogeneity of the water being sampled) or applied to grab samples. This reduces the total laboratory exposure and handling of samples to help limit unwanted contamination particularly for microplastics. However, this sampler does not facilitate the simultaneous collection of water for the analysis of dissolved pollutants. Therefore, the aim of this research was to design a new portable water sampler capable of (*1*) high volume sampling (> 10 L) and in situ isolation of particulates, (*2*) simultaneous collection of water for tyre and plastic additive chemicals analysis and (*3*) 24-h continuous monitoring. This was achieved using easily available materials with a view to make it straightforward to construct and as inexpensive as possible. Plastic-free materials were used in its construction where possible.

## Materials and methods

### Materials

All materials used for the analysis of TRWPs, paints and microplastics as well as 25 different tyre and plastic additive chemicals are provided in the Supplementary Information. For the sampler, a battery powered peristaltic pump and external battery (12 V 16 Ah) were purchased from RVA Synergies (Gloucester, UK) and Clulite (Hampshire, UK), respectively. Silicone tubing with 2 mm internal diameter was obtained from RS components (Corby, UK), and 100-mm-diameter stainless-steel woven wire sieves with apertures of 50, 100 and 250 µm were purchased from The Laboratory Store (Inverness, UK). A 90-L stainless-steel vessel (refuse bin), 90° brackets, stainless-steel circular mesh shelf (41 cm diameter) and tray (26 × 20 × 2 cm) were purchased from a local hardware store. Solder composed of tin/silver/copper (95.5/3.8/0.7) was obtained from Solders & Fluxes (Monmouthshire, UK).

### Sampling

Sampling was undertaken at the inlet of a retention pond used to manage road runoff. The pond is situated along a 22-mile stretch of a trunk road network in Aberdeen and Aberdeenshire. Runoff from approximately 5 miles of dual carriageway and 2 miles of slip roads enters the pond. The number of vehicles passing on the dual carriageway (in both directions) on an average day is ~ 37,000 (Department for Transport Road traffic statistics, 2024). The drainage system has continual flow into the pond (irrespective of rainfall) due to its length as well as inputs from infiltration. Sampling was conducted over a 24-h period from 10:30 am on 31 August 2023. No rainfall was experienced on this day with the latest rainfall (3.8 mm) occurring 3 days prior to sampling. The average flow into the retention ponds was 3.5 L/s, measured using a Micronics PF LV550 Portable Level Velocity Logger. During this time, water was continually pumped over a stack of sieves (250, 100 and 50 µm aperture sizes) at 12 mL/min (total volume sampled over 24 h was 17 L). The pump and sieves were contained within a 90-L stainless-steel vessel with lid. Upon collection, the sieves were wrapped in aluminium foil. The sieved water was mixed and an aliquot (0.01 L) collected in a glass bottle for additive analysis. The remaining water volume was measured and then discarded. Field blanks were also collected whereby a stack of sieves were left uncovered during collection of the sample sieves. Samples were transported to the laboratory within 1 h of collection.

### Analytical methods

All sample handling for particulate analysis was conducted in a HEPAire 4’ HLF B/U laminar flow hood within a clean room with supply and extract ventilation served by Air Handling Unit 10 with Variable Air Volume and inline HEPA filtering. Briefly, samples were digested with 2.7% H_2_O_2_, and density separation performed using a sodium polytungstate solution (2.0 g/cm^3^) and stained using a Rose Bengal solution to identify particles of natural composition, prior to particles being counted and characterised by Fourier transform infrared (FTIR) spectroscopy and scanning electron microscopy-energy-dispersive x-ray analysis (SEM-EDXA). Field and laboratory blanks were analysed and subtracted from sample counts (*Table S1*). Water samples for additive analysis were spiked with 1 µg/L deuterated surrogates, filtered (0.45 µm) and analysed by ultra-high-performance liquid chromatography–tandem mass spectrometry (McKenzie et al., 2024). A full description of the methods is available in the Supplementary Information.

## Results and discussion

### Design and construction of sampler

**Fig. 1:**
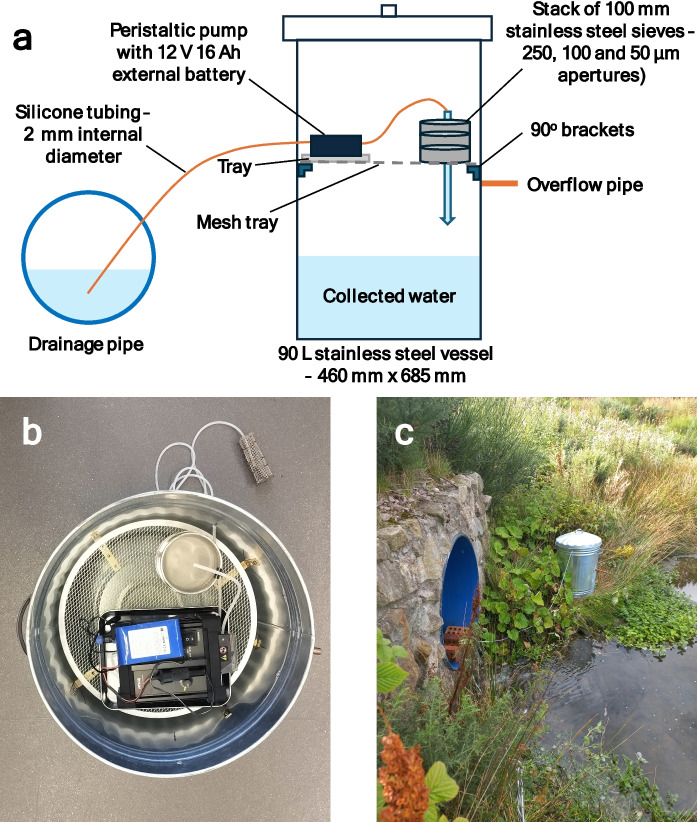
Schematic of the developed 24-h composite water sampler (**a**), aerial image of the assembled sampler with lid removed (**b**) and deployment of sampler at road drainage system entering retention pond (**c**)

The sampler was designed to simultaneously collect particulates and water separately whilst limiting the use of plastics as much as possible. Four 90° stainless-steel brackets were bolted inside the 90-L vessel at two-thirds of its height and a circular stainless-steel mesh tray fitted for the pump and sieves to be placed (Fig. 1). The pump was placed on an additional stainless-steel tray. Water was pumped through silicone tubing (2 mm internal diameter) onto a stack of sieves (250, 100 and 50 µm) with the sieved water collected in the body of the vessel. Being in direct contact with the water as it is sampled, the FTIR spectrum of the silicone tubing was obtained to ensure it did not contribute to the collected microplastic particles (see Supplementary Information *Fig, S1*). Trials using ultrapure water revealed no leaching of the studied additive chemicals from the tubing. The purchased vessel had crimped joins and was not designed to be fully watertight. To overcome this, the joins were soldered using a tin/silver/copper (95.5/3.8/0.7) alloy. An overflow pipe was also fitted below the height of the pump in case of any malfunction. Using this design, up to 50 L of water can be sampled if required.

The in situ sieving avoided the transport of a large volume of water for processing and reduced the risk of introducing laboratory contamination. A similar approach has been successfully applied to wastewater (Ziajahromi et al., 2017). Wastewater was passed through 500-, 190-, 100- and 25-µm stainless-steel mesh screens in a polyvinylchloride housing. Sampled wastewater volumes were in the range 3 to 200 L which were conducted over approximately 1 h. The same device was used in situ at a stormwater pond whereby manually collected water was passed through and the sieved water discarded (Ziajahromi et al., 2020). These studies demonstrate the suitability of such approaches applied in the field. The newly designed sampler described here can operate for ≥ 24 h whilst also collecting the sieved water to determine additive chemicals in the ‘dissolved’ phase. The use of a 16-Ah external battery pack (0.9 kg) enabled sampling to be conducted for 24 h, even in low temperatures (< 4 °C). With a total weight of 8.4 kg when ready for deployment, the sampler is easily transported and comparable to commercially available portable composite water samplers.

### Application in the field

**Fig. 2:**
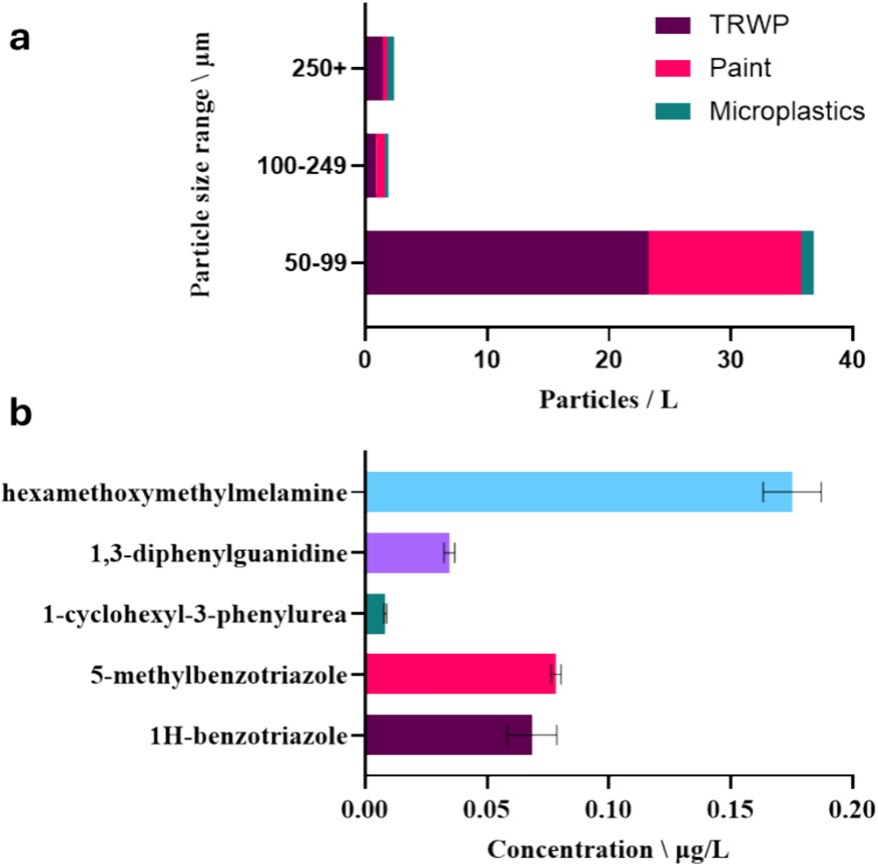
Concentrations of TRWPs, paint fragments and microplastics (> 50 µm) (**a**) and additive chemicals in water (**b**) from sampler deployed for 24-h at the inlet pipe of a roadside retention pond in North-East Scotland. Error bars represent in the standard deviation

**Fig. 3:**
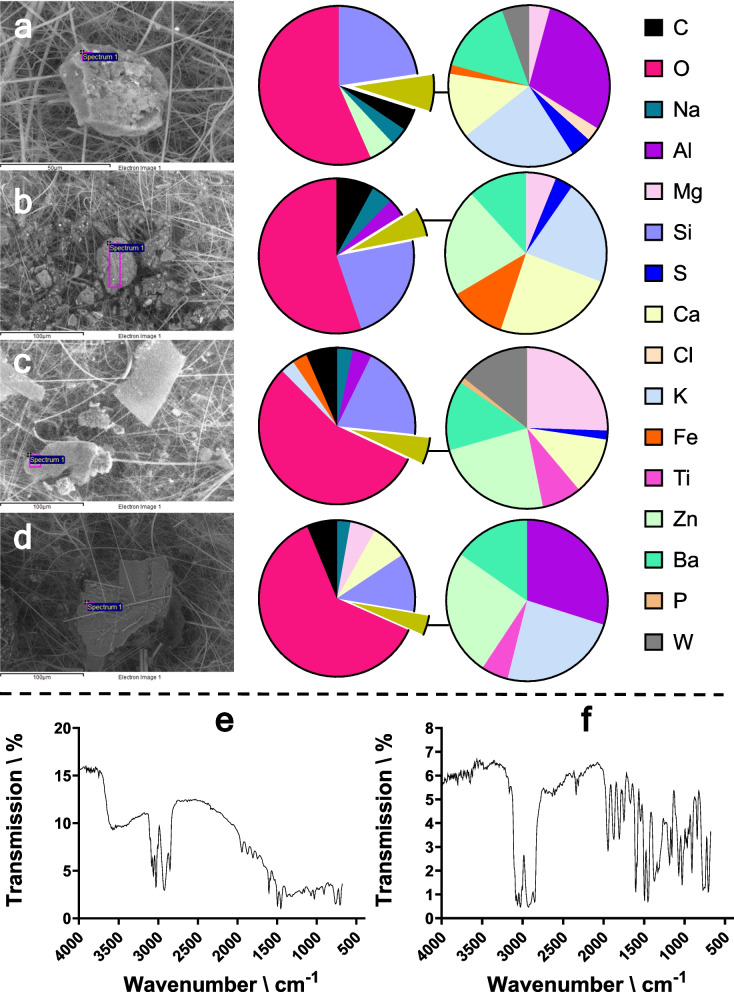
Example data for a TRWP (**a**), paint fragment (**c**) and polystyrene microplastic particle (**e**) collected during 24-h deployment of the sampler at the inlet pipe of a roadside retention pond in North-East Scotland. Corresponding reference samples of a TRWP (**b**), paint fragment (**d**) and polystyrene microplastic (**f**) are shown for comparison. Data for TRWPs and paint shows SEM images (left hand side) and elemental composition determined by EDXA (right hand side). The left pie chart shows elements with atomic % > 2.5 and the right pie chart those < 2.5%. The EDXA spectra of TRWPs and paint fragments can be found in Supplementary Information *Fig. S2*. Data for microplastics shows FTIR spectra

Application of the sampler found the number of road pollution particles > 50 µm in length followed TRWPs (25.5 particles/L) > paints (13.7 particles/L) > microplastics (2.0 particles/L) (Fig. 2). Discussion on the identification of TRWPs, paints and microplastics and their comparison to reference samples (e.g. see Fig. 3) is provided in the Supplementary Information. Most particles (89%) were within the 50–99-µm sieve size fraction. Literature data provides large variation in the size ranges of particulate pollution reported in the environment, largely due to differences in methodologies and reporting criteria between studies. Also, it has been reported that classification by sieve size alone does not directly correlate to the particle size (Ochoa et al., 2024) (i.e. some particles in the 100–249 µm sieve measured < 100 µm). Therefore, it is important to measure particle length irrespective of the sieve size for confirmation. The size reporting limit for the particle characterisation methodology applied here was 50 µm. Determination of particles < 50 µm is possible by using an appropriate analytical technique and incorporating an additional sieve with a smaller aperture size or mixing the sieved water and collecting an aliquot for subsequent analysis. No clogging of any of the different sized sieves was noted. Previous researchers have validated the use of sieves for collecting microplastics (Ziajahromi et al., 2017), but quantifying the collection efficiency of TRWPs by sieve collection remains challenging due to a lack of suitable reference material available in sufficient quantity.

Five additive chemicals were found in the collected water (Fig. 2). These included two stabilisers (1H-benzotriazole and 5-methylbenzotriazole) and two chemicals used during the vulcanisation process of tyres (hexamethoxymethylmelamine and 1,3-diphenylguanidine) as well as 1-cyclohexyl-3-phenylurea, a thermal degradation product of 1,3-diphenylguanidine. Hexamethoxymethylmelamine was found at the greatest concentration at 0.18 ± 0.010 µg/L. Previous sampling of water at the same location using grab sampling found concentrations in the range 0.039 to 0.95 µg/L, depending on the weather conditions (McKenzie et al., 2024). Elsewhere, it has been reported in similar concentrations, up to 0.24 µg/L and 0.29 µg/L in Australian surface waters (Rauert et al., 2022a, 2022b) and 0.13 µg/L in American stormwater (Hou et al., 2019). 5-Methylbenzotriazole was present at 0.078 ± 0.0020 µg/L and 1H-benzotriazole at 0.068 ± 0.010 µg/L. Both have previously been detected in environmental water samples (Hou et al., 2019; Peter et al., 2020; Rauert et al., 2022a, 2022b; Tian et al., 2020). The concentration of 1,3-diphenylguanidine was 0.034 ± 0.002 µg/L. This has previously been reported in surface waters during both wet and dry weather (Johannessen et al., 2021; Rauert et al., 2022a, 2022b; Tian et al., 2020; Zhang et al., 2023). Finally, 1-cyclohexyl-3-phenylurea was present at 0.0083 ± 0.00050 µg/L and lower than previously reported in American stormwaters and Australian surface waters (Peter et al., 2018; Rauert et al., 2022a, 2022b). Overall, the sampler was successful for simultaneous collection of particles and water to monitor road pollution.

### Cost and further considerations

Utilising the new sampler has several advantages over commercially available alternatives. Notably, it reduces plastic contact with the collected sample, enables in situ isolation of particles and is less expensive. The total cost to purchase all components was 1235 GBP (purchased during 2023, see Supplementary information *Table S2*). This is < 50% the cost of a commercially available composite sampler and in some cases < 20% depending on the manufacturer. These normally collect sub-samples of fixed volume during set time intervals normally between 15 min and 1 h during a 24-h sampling period. They collect a maximum of 5- or 10-L water. This new sampler can collect up to 50 L over 24 h (or longer) by manipulating the pump flow rate. Pumping this volume of water through the sieves was assessed, and no evidence of clogging was noted.

A drawback of the new proposed sampler is the self-assembly requirements. However, the only tools required were a small cordless drill to bore holes to fit the 90° brackets and for the pump tubing and overflow and a handheld propane torch for soldering the joins of the collection vessel. The assembly was straightforward, and the total time needed was < 1 h. It should be noted that some commercially available portable samplers have the option of cooling the collected water by packing the internal cavity of the sampler with ice or the use of cooling elements (ice packs) incorporated into their design. This may be a requirement for chemicals that are susceptible to biodegradation in collected waters. Nevertheless, the vessel could be retrofitted with an ice pack ‘jacket’ to cool the collected water.

An important consideration is that the sampler operates in a time-weighted fashion by pumping water at a constant and continuous flow. Therefore, variation in water flow and concentration of pollutants in road drainage systems experienced during storm events may demand a flow-weighted sampling process, whereby the volume of sample collected at set time intervals is proportional to the flow of water where sampling takes place. Commercial alternatives capable of true flow-weighted sampling are not readily available. Alternatively, volume-weighted samplers are, where the collection frequency of sub-samples is controlled by the flow measurements from an external flow meter (i.e. the greater the flow rate, the more often sub-samples of a fixed volume are collected). Nevertheless, this new sampler can be used to provide important information on road pollution in a time-weighted manner which is adopted in other studies (e.g. Ziajahromi et al., 2023).

## Conclusion

A new sampling approach was developed to monitor particulate and dissolved pollutants in water. It provides several benefits over commercially available composite samplers including cost, reduction of plastic use and possible contamination, mitigating the transport of large volumes of water for processing in the laboratory. The samplers ease of construction and use makes it be an attractive option for studies aimed at monitoring pollution in road runoff and in engineered systems (e.g. retention ponds). Further application is now needed to assess its performance under different monitoring scenarios such as storm events with higher particle loading.

## Data Availability

No additional datasets were generated or analysed during the current study.
